# Retracted: Chest digital dynamic radiography to detect changes in human pulmonary perfusion in response to alveolar hypoxia

**DOI:** 10.1002/jmrs.730

**Published:** 2023-10-15

The following article from *Journal of Medical Radiation Sciences*, ‘Chest digital dynamic radiography to detect changes in human pulmonary perfusion in response to alveolar hypoxia’,^1^ published online on 13 September 2022 in Wiley Online Library (http://onlinelibrary.wiley.com/), has been retracted by agreement between the journal Editor in Chief, Cherry Agustin and John Wiley and Sons, Australia. The retraction has been agreed due to unattributed overlap between Figure 3b as a conceptual figure in this article and the following article published in *Radiological Physics and Technology*, ‘Dynamic chest radiography: flat‐panel detector (FPD) based functional X‐ray imaging’ by R Tanaka, Volume 9, 2016, pages 139–153^2^. While addressed by the authors in an earlier corrigendum, further editorial review of the overlap, the permission received, and the article submission details have resulted in a decision to retract in line with the journal's editorial policies.
